# Epidemiology of Congenital Hypothyroidism in Markazi Province, Iran

**DOI:** 10.4274/jcrpe.1287

**Published:** 2014-06-05

**Authors:** Fatemeh Dorreh, Parsa Y. Chaijan, Javad Javaheri, Ali Hossein Zeinalzadeh

**Affiliations:** 1 Arak University of Medical Sciences, Thyroid Disorders Research Center, Department of Pediatrics, Arak, Iran; 2 Tabriz University of Medical Sciences, National Public Health Management Center, Department of Community Medicine, Tabriz, Iran

**Keywords:** congenital hypothyroidism, Epidemiology, Neonatal screening, Iran

## Abstract

**Ob­jec­ti­ve:** The aim of this study was to investigate the epidemiology of congenital hypothyroidism (CH) among newborns in Markazi Province, Iran.

**Methods:** This cross-sectional study was conducted from 2006 to 2012. Blood samples were taken between 3 to 5 days after birth from the heel. Thyroid stimulating hormone (TSH) was tested using the enzyme-linked immunosorbent assay method and was employed as the screening test. Newborns with abnormal screening results (TSH >5 mIU/L) were re-examined. The data were analyzed using SPSS.

**Results:** A total of 127 112 infants were screened. Of these, 51.2% were male and 48.8% were female. The coverage rate of the screening program was 100%. Of 6102 recalled subjects (re-call rate 4.8%), 414 cases with CH were detected, yielding a CH prevalence of 1:307 (female:male ratio 1:0.95). The prevalence of permanent and transient CH was 1:581 and 1:628, respectively.

**Conclusion:** This study reveals that the prevalence of CH is higher compared to worldwide levels. Comprehensive and complementary studies for recognizing related risk factors should be a priority for health system research in this province.

## INTRODUCTION

Congenital hypothyroidism (CH) is reported to affect one in 2000 to 4000 newborns, making it one of the most common causes of preventable mental retardation (1). Neonatal screening programs for CH allow early effective diagnosis and treatment of the condition (2). The CH patients may have a developmental abnormality such as thyroid gland dysgenesis or thyroid dyshormonogenesis (3). The prevalence of CH has been shown to vary among different populations around the world. The first screening programs for CH were conducted in North America in 1972 (4). Studies have suggested that the prevalence of CH appears to be on the increase in many countries due to many factors such as diagnosis of mild cases, increasing maternal age, increasing premature birth, multiple pregnancies and lower cut-off point (1,5,6,7). In Iran, screening programs for CH were first carried out by Azizi et al (8) in Tehran in 1987. The first screening for CH in in Markazi Province was performed in 2006. According to the national screening program, the prevalence of CH in Iran is higher than global statistics and different prevalence figures are reported in different provinces (5,6,9,10,11).

The purpose of this study was to investigate the epidemiology of CH among neonates screened in Markazi Province, Iran, in the years 2006-2012.

## METHODS

In this descriptive cross-sectional study, we used the data collected from the CH screening of 127 112 neonates from all sources (private or public) in Markazi Province, Iran, from August 2006 to August 2012. All neonates were screened according to the standard CH screening protocol. In accordance with the design of the screening program, samples were collected from all 10 cities of the province. Heel prick blood samples were taken on 903 Whatman filter papers by trained nurses, mostly within 3-5 days of birth. They were dried and immediately transferred to the screening laboratory of the province by express mail service. Thyroid stimulating hormone (TSH) was tested using the enzyme-linked immunosorbent assay (ELISA) method.

In accordance with the national screening program protocol, neonates were re-called based on the level of the first TSH measurements. TSH ≥5 mIU/L in neonates aged 3-7 days and TSH ≥4 mIU/L in infants aged greater than eight days were considered abnormal. For such cases, additional confirmation tests [thyroxine (T4), TSH and triiodothyronine resin uptake (T3RU)] were performed. In addition, if the initial TSH level was ≥20 mIU/L, treatment was initiated and thyroid function tests were performed concomitantly. If the results of the second set of tests were within normal limits, the neonate was considered as a case of transient TSH elevation and treatment was halted. All the re-called neonates were examined clinically by a pediatrician, a focal point of program. According to the results of the second measurement performed between 7 and 28 days of birth, neonates were considered as hypothyroid if T4 was <6.5 µg/dL and TSH was >10 mIU/L (12). Thus, in both premature and full-term neonates whose T4 measurements were low according to their weight (13), complementary tests including T3RU and free T4 index had been performed and treatment started if the results were abnormal. Neonates with confirmed hypothyroidism underwent treatment with a single daily dose of levo T4 (LT4) (10-15 µg/kg/day). Infants diagnosed with CH were followed closely in the first three years of life. They were followed up monthly or every two months during the first year of life and every two to three months during the second and third years.

In order to distinguish between permanent and transient cases of CH, LT4 therapy was discontinued for 4 weeks in children who were ≥3 years old, after which time thyroid function tests (T4 and TSH) were evaluated by the same laboratory methods and the same enzymatic kits.

Thyroid scintigraphy and/or ultrasonography were performed in all patients after discontinuing treatment. If the thyroid function tests showed a high TSH with low T4, the patient was diagnosed to have permanent CH (12,13). The etiology of CH among patients with permanent CH was determined by both scintigraphic and ultrasonographic imaging for thyroid dysgenesis (agenesis, ectopia and hypoplasia) and thyroid function tests. Patients with abnormal thyroid function test results but normal scintigraphy and ultrasonography scans were considered to be cases of dyshormonogenesis.

Variables such as gender, residency (lodging), birth weight, maturity (full term or premature), birth season, screening sampling age, familial (maternal) history of thyroid disorder, treatment initiation age and parental consanguinity were recorded. 

**Laboratory Methods**

TSH was measured by ELISA using Iran Padtan Elm Company kits. The sensitivity of TSH test was 0.5 mIU/L. The accuracy of the TSH test was assessed using both intra- and inter-assay results. Intra-assay coefficients of variation at TSH concentrations of 17.7, 78.4 and 162.4 mIU/L were 8.6%, 9.8% and 9.1%, respectively. The inter-assay coefficients of variation for different methods at TSH concentrations of 15, 26.5 and 54.9 mIU/L were 11.3%, 9% and 8.8%, respectively.

**Statistical Analysis**

The data were analyzed using SPSS software. Frequency, mean and standard deviation for demographic data and TSH levels in neonates were estimated. Qualitative variables were compared using the chi-square test and quantitative/qualitative variables were compared by t-test and Kruskal-Wallis tests. All p-values were two-sided with p<0.05 being considered significant. The Medical Ethics Committee of Arak University of Medical Sciences approved the study protocol.

## RESULTS

During the study period, 127 112 neonates were screened. Of the screened newborns, 65 090 (51.2%) were male and 62 022 (48.8%) were female. The female:male ratio was 1.00/0.95. Except for year 2006, approximately 100% of all neonates in the population were screened. In 79.9% of neonates, the samples were taken 3-5 days after birth. In 18.9% of the neonates, the samples were taken between 5 and 21 days and in 1.1% - later than 21 days after birth.

The distribution of serum TSH values among the hypothyroid neonates is shown in Table 1. The mean TSH level in all measured samples was 19.1±29.1 mIU/L. The distribution of TSH levels showed that 372 (89%) of 414 diagnosed CH neonates had initial TSH values over 5 mIU/L. In addition, 215 (52%) had TSH values between 4.5 and 9.9 mIU/L. In accordance with the national guidelines, these neonates were re-called for additional confirmatory testing. Overall, 6102 neonates (4.8%) were re-called. Of these, 414 (6.8%) were diagnosed as CH cases. The prevalence of CH among all subjects studied was 3.25/1000 or 1:307 live births. Among neonates with CH, 212 (51.2%) were male and 202 (48.8%) were female (male: female ratio 1.05:1). The prevalence of CH among male and female neonates was 3.15/1000 and 3.25/1000, respectively. Chi-square test showed no statistically significant relation between gender and CH (p=0.236). The mean birth weight of the total group of CH neonates was 3047±60g. This figure was 3188±23 and 1905±75 grams for term and preterm CH neonates, respectively. There was no statistically significant difference between mean birth weight of neonates and hypothyroid subjects (p=0.34). Of the CH cases, 360 (89.1%) were full-term and 44 (10.9%) were premature. There was no statistically significant difference between maturity and hypothyroidism (p=0.13). In 22 of CH neonates (5.3%, 22/414), the mother had a history of thyroid disease. Also, in 58 (14%, 58/414) cases, there was a family history of thyroid disease. There was a statistically significant relation between family history of thyroid diseases and hypothyroidism (p=0.037). Parental consanguinity was present in 26% (105/414) of all CH cases. In 76 (18.5%) cases, parents had third-cousin consanguinity and in 29 (7%), there was fourth -cousin consanguinity between parents. There was no statistically significant difference between parental consanguinity and hypothyroidism (p=0.456).

The frequency of CH varied according to season. The highest prevalence of CH was in autumn, but there was no relation between CH and season because this difference was not statistically significant (p=0.094). 

Table 2 shows the characteristics of neonates with permanent and transient CH. Of 414 patients diagnosed with hypothyroidism, a diagnostic discontinuation of therapy at around three years was undertaken in 235; in these 235 patients, treatment was discontinued for 4 weeks and their T4 and TSH were measured again. Of these, 122 (51.9%) were found to have permanent CH. The remaining 113 (48.1%) were diagnosed as transient CH. Of 127 112 screened neonates, 71019 had reached 3 years or more and in this group, the prevalence of permanent and transient CH was found as 1:581 (1.72/1000) and 1:628 (1.59/1000) live births, respectively. Of the CH neonates, sonography was performed in 7 and all were assessed as normal (2 cases of transient CH and 5 cases of permanent CH). Thyroid scintigraphy was performed in 26% (61/235) of hypothyroid neonates. According to scintigraphic findings performed in 43 permanent and 18 transient hypothyroid neonates, all transient CH patients had normal thyroid glands and in permanent CH patients, 5 (2.1%) had thyroid dysgenesis (2 agenesis or hypoplasia and 3 ectopia) and 6 (2.55%) had dyshormonogenesis. Overall, in 50 (21.3%) neonates, the thyroid scan was normal. 

## DISCUSSION

The prevalence of CH among neonates in Markazi Province of Iran has not previously been determined, but this study shows that the estimated prevalence is one in 307 live births. This is approximately 6.5 to 13-fold of figures reported from other countries (1). With more experience from state, regional and national screening programs, it has become apparent that the prevalence of CH varies throughout the world, but the worldwide reported prevalence is one in 2000-4000 live births (1,14). According to recent data, this prevalence has been reported as 1:469 in Turkey (15), 1:800 in Greek Cypriot populations (16) and 1:2372 in the United States (17). There are a few pilot studies on the prevalence of CH conducted in Iran prior to the National Screening Program. Ordookhani et al (18) reported that the prevalence of CH in Tehran (Iran’s capital) was 1:914 in 20107 newborns screened between 1987 and 2001. The estimated prevalence in our study was significantly higher than that found in the previous studies (1 in 1465, 1 in 500 and 1 in 666 live births in Fars, the eastern and northwestern parts of Iran, respectively) (9,11). 

Differences in the prevalence of CH reported from different parts of the world may be due to several factors. The studies have reported a high CH prevalence in Iran. Iodine deficiency is a known risk factor for CH (8). The wide variability of CH prevalence with the same methodology in different parts of the same country may reflect the degree of iodine deficiency in each region, but this problem has been eradicated in Iran (19). However, there may be factors other than iodine deficiency affecting CH incidence in different regions. The differences in incidence may be due to differences in ethnic, environmental, genetic and autoimmune factors (20,21,22,23). Thus comprehensive, complementary and multicenter studies for recognizing the relevant factors affecting the prevalence of CH are among the priorities of the health system research in Iran.

CH re-call rates also vary (from 0.16% to 3.3%) among different countries in which the screening programs were performed between 3 and 5 days after birth (24,25,26). In the present study, the recall rate was 4.8%, which was a comparatively higher figure. The difference can be due to the sampling method, a different way of performing laboratory tests, or may be a reflection of the degree of iodine deficiency in each region (25,27). However, this study was preliminary and further studies are needed to clarify the causes of these differences. 

According to the American Academy of Pediatrics recommendations, the optimal testing time is between 48 hours and 4 days of birth. The national CH screening programs in Iran aim to test neonates between three and five days of life. However, the data indicate that not all, but approximately 80% of the samples in our study were taken between the third and fifth days of life. Age at the start of treatment has been demonstrated to be an important determinant of neurodevelopmental outcome and early newborn screening and early treatment with T4 may prevent the adverse neurodevelopmental consequences of delayed diagnosis and treatment (28,29). However, in most countries, treatment is now started earlier, usually being initiated within the first 2 weeks of life (30,31). In our study, mean age of initiation of treatment was 22.7±11 days. We hope that over the years, there will be a trend for earlier start of treatment. 

The female:male ratio for CH varies in different countries and is reported as 6:1 in Estonia, 3:1 in Saudi Arabia, 1.2:1 in Japan and as 1.4:1 in the eastern part of Iran (15,32,33,34). Results of nearly all screening programs also suggest a female preponderance for CH, approaching a 2:1 female to male ratio (1). In the present study, this ratio was 1:0.95. Recently, Zeinalzadeh and Talebi reported the results of screening for CH in 62459 infants in East Azerbaijan, Iran. Female/male ratio was 1:1.4 (11). The differences in prevalence and in female/male ratios may be due to differences in prevalence of consanguineous marriages and to differences in frequency of undiagnosed family history of CH, as reported also by Castanet et al (20). Again, the preponderance of CH in females might be due to undiscovered genetic factors, perhaps linked to autoimmuity, which is usually more common in females (1). In 22 of the CH neonates in our study (5.3%), the mother also had a history of thyroid disease, supporting the importance of genetic factors as a possible cause of CH (16).

Seasonal variation in CH incidence has been reported in some studies (3,11,15). These studies have suggested that the causes of CH are not only intrinsic factors such as immune system dysfunction or gene defects, but environmental factors also play a role. Moreover, there was some speculation as to a possible seasonal variation in the incidence of CH. In our study, the highest prevalence was found in autumn and the lowest prevalence in spring. However, we agree that these results are not conclusive and that this topic is still under debate (1,34).

Studies suggest that children with transient CH require a lower dose of T4 in order to maintain normal thyroid hormone levels than those with permanent CH (16). These findings are not in agreement with the results from our study because there was no statistically significant difference between the two groups. However, mean TSH levels before treatment were significantly higher in the patients with permanent CH than in those with transient CH. This finding indicates that the initial TSH level obtained at screening may have a predictive role for identifying the permanent forms of CH from the transient ones. The results of a study performed in Brazil by Silva et al (35) to assess the characteristics and etiologies of transient CH, the TSH initial levels were reported not to be relevant to determine whether the thyroid dysfunction was transient or permanent. In our study, the prevalence of permanent CH was high with a prevalence rate of 1 in 581 live births. The prevalence of transient CH was 1 in 628 live births. Prevalence of permanent CH was reported to be 1 in 2679 in Quebec-Canada, 1 in 2418 in China and 1 in 1800 in Greece (16,36,37). The higher prevalence of CH in Arak was due to a higher rate of permanent CH, whereas in other studies from Iran, the rate of transient CH is higher than that of permanent CH. These differences may be due to differences in screening method or to differences in environmental or immunologic factors. To our knowledge, there have been no systematic studies in these fields among newborns with transient versus permanent hypothyroidism in Arak. 

In the present study, parental consanguinity was found in 19 (15.8%) and 8 (7.2%) cases of permanent and transient CH, respectively, indicating that parental consanguinity was 2.2 times more frequent in cases of permanent CH compared to neonates with transient CH. Other studies in Iran have also shown that parental consanguinity is higher in cases of CH than among neonates without CH (38). Further investigations are needed to evaluate the role of parental consanguinity and its effect on the incidence of CH and whether it has a greater effect on the incidence of permanent versus transient CH.

In conclusion, the results of this study reveal that the prevalence of CH is higher in the Markazi Province of Iran than the figures reported from other parts of the world. The study also shows the need to integrate proper screening programs for CH and also to initiate programs to evaluate the related risk factors in the routine healthcare system in this province.

**Acknowledgements**

Our study was funded by the Thyroid Research Center of Arak University of Medical Sciences & Health Services. There is no conflict of interest in this manuscript (real or perceived) and there was no study sponsor(s) in all process of the study.

## Figures and Tables

**Table 1:**
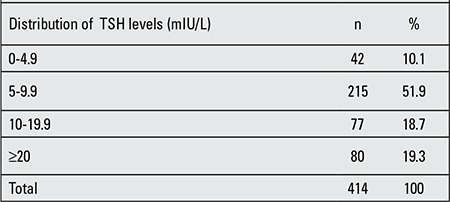
Distribution of thyroid stimulating hormone (TSH) levels among hypothyroid neonates, on their first measurement

**Table 2:**
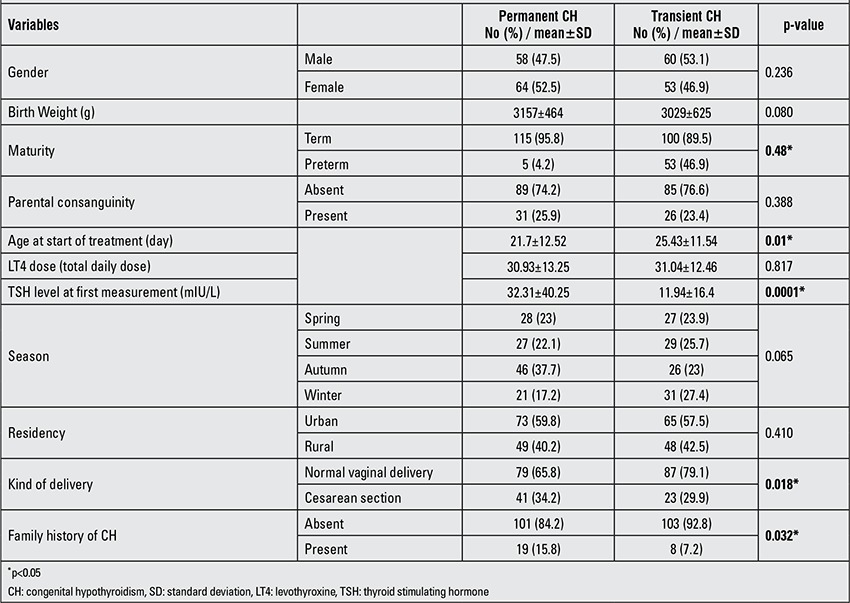
Univariate analysis of characteristics of the neonates with permanent and transient CH
